# Systematic Analysis Uncovers Associations of PGK1 with Prognosis and Immunological Characteristics in Breast Cancer

**DOI:** 10.1155/2021/7711151

**Published:** 2021-11-08

**Authors:** Liangdong Li, Yang Bai, Yang Gao, Deheng Li, Lei Chen, Changshuai Zhou, Mingtao Feng, Xin Chen, Wei Jin, Yiqun Cao

**Affiliations:** ^1^Department of Neurosurgery, Fudan University Shanghai Cancer Center, Shanghai 200032, China; ^2^Department of Oncology, Shanghai Medical College, Fudan University, Shanghai 200032, China; ^3^Department of Obstetrics and Gynecology, The Affiliated Obstetrics and Gynecology Hospital of Fudan University, Shanghai 200030, China; ^4^Department of Breast Surgery, Key Laboratory of Breast Cancer in Shanghai, Fudan University Shanghai Cancer Center, Shanghai 200030, China

## Abstract

**Objective:**

Phosphoglycerate kinase 1 (PGK1) is an essential enzyme in the process of glycolysis and mitochondrial metabolism. Herein, we conducted a systematic analysis to uncover the clinical implication of PGK1 deregulation in breast cancer.

**Methods:**

Expression pattern and prognostic significance of PGK1 were comprehensively assessed across pan-cancer based on RNA-seq profiles from the TCGA project. Associations of PGK1 with immunological features in the tumor microenvironment (immune checkpoints, immune response predictors (tumor mutation burden (TMB) and microsatellite instability (MSI)), and tumor-infiltrating immune cells) were systematically analyzed. The role of PGK1 in the prediction of breast cancer prognosis was also evaluated. GSEA was presented for investigating biological pathways involved in PGK1.

**Results:**

PGK1 was specifically overexpressed in most of cancer types, including breast cancer. High PGK1 expression was indicative of undesirable overall survival, progression-free interval, disease-specific survival, and disease-free interval for various cancers. Furthermore, high PGK1 levels exhibited prominent correlations to immune checkpoints and high response to immunotherapy across pan-cancer. Notably, ROC curves confirmed that PGK1 can robustly predict breast cancer prognosis. Furthermore, PGK1 might shape an inflamed tumor microenvironment following the evidence that PGK1 was positively correlated to the abundance levels of tumor-infiltrating immune cells such as CD8+ T cell and NK cell in breast cancer. GSEA results revealed that PGK1 participated in metabolism and carcinogenic pathways.

**Conclusion:**

Collectively, PGK1 was capable of robustly predicting the prognosis and response to cancer immunotherapy in breast cancer.

## 1. Introduction

Breast cancer represents the dominating cause of cancer-related deaths among women [1]. This malignancy is mainly classified as Normal-like, Luminal A, Luminal B, HER2-enriched, and Basal-like subtypes. Surgery, systemic treatment such as chemotherapy, hormonal therapy, and targeted therapy, is selected in line with the molecular features to combat this malignancy [2–4]. However, many patients cannot benefit from conventional treatment, leading to undesirable survival outcomes. The heterogeneity of breast cancer biology presents a profound challenge to personalized therapy [5]. Immune checkpoint inhibitors (ICIs) have generated durable clinical remissions in several cancer types [6–8]. Several clinical trials of ICIs have focused on the effects of CTLA4 and PD1/PDL1 inhibitors on breast cancer [6–8]. Nevertheless, these ICIs are less effective as a single agent in breast cancer, partly due to low infiltration levels of tumor-infiltrating lymphocytes [9]. Tumor immune evasion and high heterogeneity contribute to the disappointing outcomes [10]. Strategies to improve immune response in breast cancer through combination with chemotherapy or targeted therapies are urgently required to prolong the survival duration of patients [11]. For instance, PD-L1 inhibitor combined with chemotherapy has been approved for metastatic triple-negative breast cancer [12]. Tumor-infiltrating lymphocytes participate in modulating chemotherapeutic responses and their presence ameliorates survival outcomes of breast cancer [13]. Hence, comprehensive assessment of tumor-infiltrating lymphocytes and their specific modulators is capable of guiding prognosis as well as appropriate sequencing of treatment in breast cancer.

Most of tumor cells exhibit increased glycolysis as well as reduced mitochondrial metabolism, called as Warburg effect [14]. This phenomenon has become a promising therapeutic target against cancer. Phosphoglycerate kinase 1 (PGK1) is the first ATP-producing enzyme in the glycolytic process [15]. This enzyme catalyzes the transformation of 1,3-diphosphoglycerate to 3-phosphoglycerate, thereby producing one molecule of ATP [16]. Increasing evidence suggests the prominent upregulation of PGK1 as an oncogene in various cancer types [17–19]. For instance, O-GlcNAcylation of PGK1 may coordinate glycolysis and TCA cycle to enhance tumor growth [20]. Hypoxia-mediated acetylation of PAK1 promotes autophagy as well as brain tumorigenesis through phosphorylation of ATG5 [21]. Nevertheless, the role of PGK1 in breast cancer is needed to be thoroughly investigated. Herein, we conducted a pan-cancer analysis of the expression pattern and immunological features of PGK1. Moreover, PGK1 might shape an inflamed tumor microenvironment in breast cancer as well as possesses the potential to estimate breast cancer prognosis.

## 2. Materials and Methods

### 2.1. Analysis of Tumor Immune Estimation Resource (TIMER) Database

TIMER2.0 database (http://timer.cistrome.org/) represents an integrated resource that provides gene expression and immune infiltration analyses across 33 cancer types [22]. TIMER2.0 web server may enable users to compare a gene in tumor with normal specimens across diverse cancers on the basis of the expression profiles of the Cancer Genome Atlas (TCGA) [23]. Also, this web platform estimates the abundance levels of six immune infiltrates (including B cell, CD4+ T cell, CD8+ T cell, neutrophil, macrophage, and dendritic cell) based on the TIMER algorithm. Here, the TIMER2.0 web server was employed for analyzing the differential expression of PGK1 in tumor and normal tissue specimens in diverse cancers. The associations between mRNA expression of PGK1 and abundance of six lymphocytes were evaluated across breast cancer samples through Spearman correlation analysis. Moreover, this study assessed the correlations of PGK1 with immune checkpoints of 16 immune cells at the mRNA levels across different cancers. The mRNA expression of PGK1 was expressed as log2 Transcripts Per Million (TPM) value.

### 2.2. Prognostic Analysis of PGK1 across Pan-Cancer

Level 3 RNA-seq as well as matched follow-up data for 33 cancer types was acquired from the TCGA database via Genomic Data Commons (GDC). Univariate-cox regression analyses were presented for evaluating the correlations of PGK1 mRNA expression with clinical outcomes of 33 cancer types. Hazard ratio (HR), 95% confidence interval (CI), and *p* value were calculated through forestplot package. Kapan-Meier curves were conducted for investigating the correlations of PGK1 with overall survival (OS), disease-free interval (DFI), disease-free survival (DSS), and progression-free interval (PFI) across pan-cancer samples with log-rank test utilizing survival and survminer packages.

### 2.3. Correlation between PGK1 and Immune Checkpoints

Spearman correlation between PGK1 and immune checkpoints including BTLA, CD200, TNFRSF14, NRP1, LAIR1, TNFSF4, CD244, LAG3, ICOS, CD40LG, CTLA4, CD48, CD28, CD200R1, HAVCR2, ADORA2A, CD276, KIR3DL1, CD80, PDCD1, LGALS9, CD160, TNFSF14, IDO2, ICOSLG, TMIGD2, VTCN1, IDO1, PDCD1LG2, HHLA2, TNFSF18, BTNL2, CD70, TNFSF9, TNFRSF8, CD27, TNFRSF25, VSIR, TNFRSF4, CD40, TNFRSF18, TNFSF15, TIGIT, CD274, CD86, CD44, and TNFRSF9 was assessed across pan-cancer.

### 2.4. Correlation between PGK1 and Tumor Mutation Burden (TMB) and Microsatellite Instability (MSI)

TMB, the whole number of somatic coding mutations within a tumor, represents an emerging biomarker of sensitivity to ICIs [24]. MSI, a molecule fingerprint of defects within the mismatch repair system, represents another predictor to guide immunotherapy [25]. Associations of PGK1 expression with TMB and MSI were analyzed with Spearman correlation analysis across pan-cancer.

### 2.5. Expression and Prognostic Significance of PGK1 in Breast Cancer

PGK1 mRNA expression was compared between 1097 breast cancer and 572 normal tissues in the TCGA cohort with the Wilcoxon test. In line with the median value of PGK1 mRNA expression, we clustered breast cancer subjects into high as well as low expression subgroups. For evaluating the prognostic implication of PGK1, survival analyses were presented. Survival difference was estimated between two groups with log-rank tests. Receiver operator characteristic (ROC) curve was drawn for investigating the predictive performance of PGK1 expression. Area under the curve (AUC) of one-, three-, and five-year survival was determined. Gene Expression Profiling Interactive Analysis 2 (GEPIA2; http://gepia2.cancer-pku.cn/) web server offers gene expression analysis in tumor and normal specimens from the TCGA and Genotype-Tissue Expression (GTEx) projects [26]. PGK1 expression was compared among different stages of breast cancer using the GEPIA2 tool.

### 2.6. Analysis of Association between PGK1 and Clinical Phenotype

Sankey diagram was conducted for evaluating the correlations of PGK1 with clinical phenotypes (pathological T stage (T1-4), pathological N stage (N0-3), pathological M stage (M0 and M1), and survival status (alive and dead)) of breast cancer patients in TCGA dataset utilizing ggalluvial package.

### 2.7. Estimation of Immune Infiltrates

Immunedeconv package [27] provides an access to six algorithms to reliably quantify the abundance of lymphocytes from bulk RNA-seq profiles, including Microenvironment Cell Populations-counter (MCP-counter) [28], quanTIseq [29], Cell type Identification By Estimating Relative Subsets Of RNA Transcripts (CIBERSORT) [30], xCell [31], and TIMER [22].

### 2.8. Gene Set Enrichment Analyses (GSEA)

GSEA provides a robust way to analyze molecular profiling data. To evaluate biological pathways involved in PGK1, breast cancer samples were divided into high and low PGK1 subgroups. Afterward, enrichment score (ES) of Kyoto Encyclopedia of Genes and Genomes (KEGG) pathways and hallmarks of cancer was calculated utilizing GSEA software (version 4.1.0) [32]. KEGG gene sets and hallmark gene sets were curated from Molecular Signature Database (MSigDB; version 3.0; http://www.broadinstitute.org/msigdb) that offers the most extensive gene sets for carrying out GSEA [33]. Significance level of ES was estimated with empirical phenotype-based permutation test. Estimated significance was corrected through multiple hypothesis testing. The ES was normalized for each gene set to yield a normalized enrichment score (NES) and false discovery rate (FDR) that corresponded to each NES was determined through comparison of the tail of the investigated and null distributions for the NES.

### 2.9. Statistical Analysis

All analysis was presented with R software (version 4.0.3; R Foundation for Statistical Computing, Vienna, Austria). Qualitative variables were analyzed utilizing Fisher's exact test. Quantitative variables were analyzed with Student's *t* or Wilcoxon tests. Pearson or Spearman correlation analyses were utilized for evaluating the correlation between two variables. *p* < 0.05 was indicative of statistical significance.

## 3. Results

### 3.1. Pan-Cancer Analysis of Expression and Prognostic Impacts of PGK1

**Figure 1:**
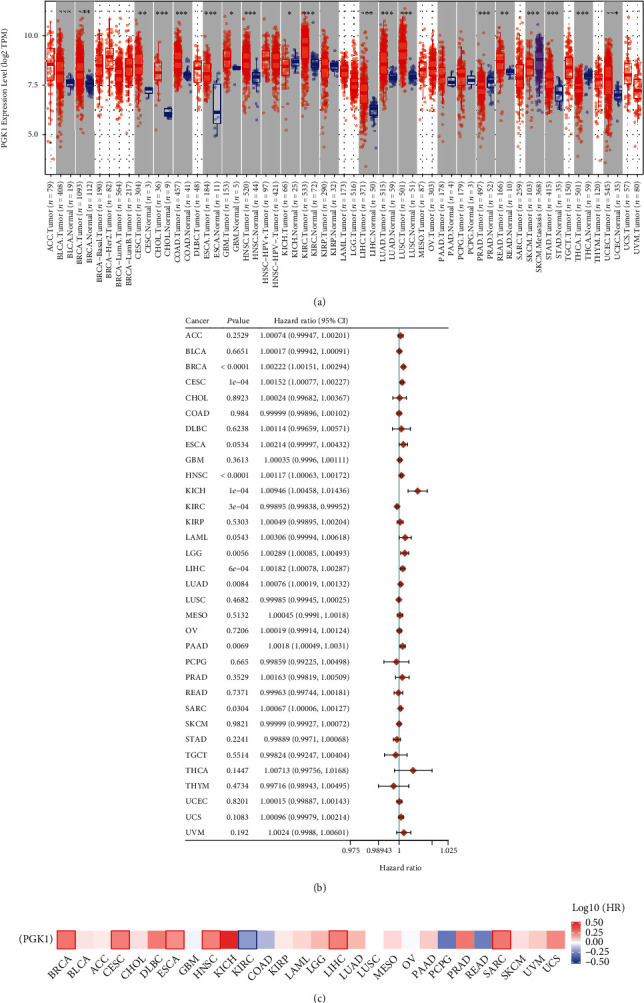
Expression and prognostic impacts of PGK1 across pan-cancer. (a) Abnormal expression of PGK1 mRNA between control and cancer tissue specimens based on the TIMER2.0 web server. The red, blue, and purple bubbles separately meant tumor, normal, and metastatic specimens. ^∗^*p* < 0.05; ^∗∗^*p* < 0.01; ^∗∗∗^*p* < 0.001. (b) Univariate-cox regression analysis for evaluating the correlation between PGK1 mRNA expression and survival outcomes of cancer patients. Forest plots showed the hazard ratio (HR), 95% confidence interval (CI), and *p* of PGK1 in 33 cancer types. (c) Prognostic impacts of PGK1 mRNA expression across pan-cancer utilizing the Survival Map module of the GEPIA2 web server. The heat map showed the HRs in logarithmic scale (log10) of PGK1. The red and blue blocks separately denoted increased and reduced risks. The rectangle with frame meant the significantly undesirable and desirable clinical outcomes according to prognostic analysis.

**Figure 2:**
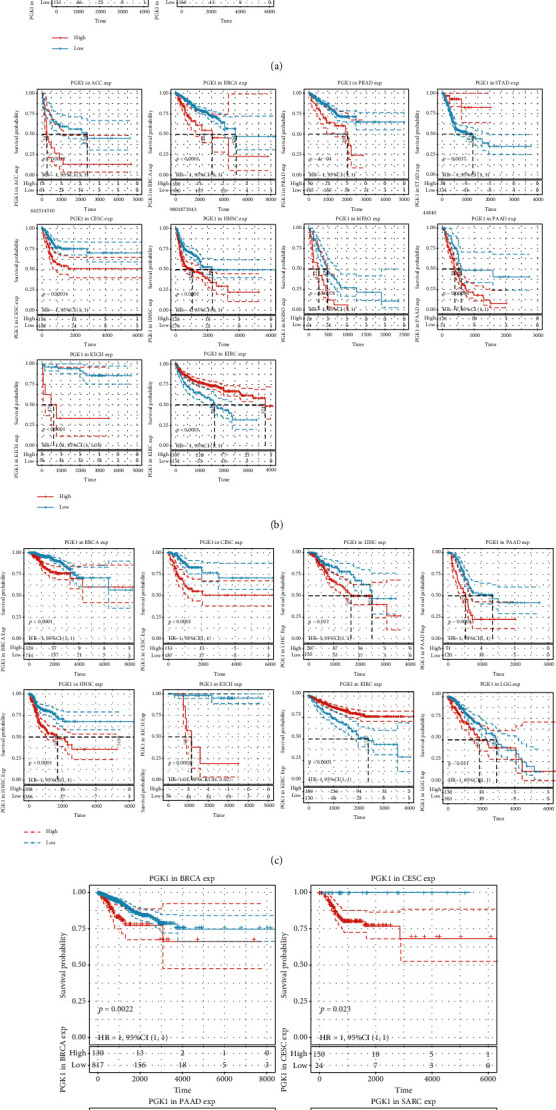
Impact of PGK1 expression on overall survival (OS), progression-free interval (PFI), disease-specific survival (DSS), and disease-free interval (DFI) across pan-cancer. (a) Kaplan-Meier curve depicting the marked difference in OS between up- and downregulated PGK1 samples in diverse cancer types. (b) Kaplan-Meier curve demonstrating the marked difference in PFI between up- and downregulated PGK1 samples in different cancer types. (c) Kaplan-Meier curve depicting the marked difference in DSS between up- and downregulated PGK1 samples in diverse cancer types. (d) Kaplan-Meier curve depicting the marked difference in DFI between up- and downregulated PGK1 samples in diverse cancer types. *p* value was determined with log-rank test.

Through the TIMER2.0 web server, this study presented the differential expression of PGK1 in normal and tumor tissue specimens across pan-cancer. We observed that PGK1 mRNA expression exhibited the prominent upregulation in BLCA, BRCA, CESC, CHOL, COAD, ESCA, GBM, HNSC, KIRC, LIHC, LUAD, LUSC, READ, STAD, and UCEC tissues in comparison to controls (Figure 1(a)). Also, its expression was markedly downregulated in KICH, PRAD, and THCA tissues than normal tissues. Higher mRNA expression of PGK1 was found in SKCM metastasis than primary SKCM. Univariate-cox regression analysis uncovered that PGK1 upregulation predicted undesirable survival outcomes of patients with BRCA (*p* < 0.0001; HR: 1.00222 (1.00151-1.00294)), CESC (*p* = 1*e* − 04; HR: 1.00152 (1.00077-1.00227)), HNSC (*p* < 0.0001; HR: 1.00117 (1.00063-1.00172)), KICH (*p* = 1*e* − 04; HR: 1.00946 (1.00458-1.01436)), LGG (*p* = 0.0056; HR: 1.00289 (1.00085-1.00493)), LIHC (*p* = 6*e* − 04; HR: 1.00182 (1.00078-1.00287)), LUAD (*p* = 0.0084; HR: 1.00076 (1.00019-1.00132)), PAAD (*p* = 0.0069; HR: 1.0018 (1.00049-1.0031)), and SARC (*p* = 0.0304; HR: 1.00067 (1.00006-1.00127); Figure 1(b)). In contrast, PGK1 downregulation was predictive of unfavorable prognosis of KIRC (*p* = 3*e* − 04; HR: 0.99895 (0.99838-0.99952)). Through the Survival Map module of the GEPIA2 web server, we observed that PGK1 was a risk factor of BRCA, CESC, ESCA, HNSC, LIHC, and SARC prognosis while PGK1 was a protective factor of KIRC prognosis (Figure 1(c)). We also investigated the influence of PGK1 expression on OS, PFI, DSS, and DFI across pan-cancer. High PGK1 expression markedly predicted poorer OS of BRCA (*p* < 0.0001), CESC (*p* < 0.0001), HNSC (*p* < 0.0001), KICH (*p* < 0.0001), LGG (*p* = 0.0027), LIHC (*p* < 0.0001), LUAD (*p* < 0.0001), PAAD (*p* = 0.00026), and SARC (*p* < 0.0001) but its upregulation was in relation to favorable OS of KIRC (*p* < 0.0001; Figure 2(a)). As shown in Figure 2(b), PGK1 upregulation was indicative of unfavorable PFI for ACC (*p* = 0.0018), BRCA (*p* < 0.0001), CESC (*p* = 0.00034), HNSC (*p* < 0.0001), KICH (*p* < 0.0001), MESO (*p* = 0.00078), PAAD (*p* = 0.00096), and PRAD (*p* = 4*e* − 04) but its upregulation indicated favorable PFI for patients with KIRC (*p* < 0.0001) and STAD (*p* = 0.0037). In Figure 2(c), we observed that high PGK1 expression was in relation to undesirable DSS of patients with BRCA (*p* < 0.0001), CESC (*p* < 0.0001), HNSC (*p* < 0.0001), KICH (*p* < 0.0001), LGG (*p* = 0.011), LIHC (*p* = 0.012), and PAAD (*p* = 0.00031). In contrast, low PGK1 expression contributed to undesirable DSS for KIRC (*p* < 0.0001). The differences in DFI were also evaluated between high and low PGK1 groups. As a result, PGK1 upregulation displayed prominent associations with poorer DFI for BRCA (*p* = 0.0022), CESC (*p* = 0.023), PAAD (*p* = 0.0022), and SARC (*p* = 0.0081; Figure 2(d)). Collectively, PGK1 exerted a carcinogenic role in most of cancers, especially breast cancer.

### 3.2. Association between PGK1 and Immune Checkpoint, TMB, and MSI in Pan-Cancer

**Figure 3:**
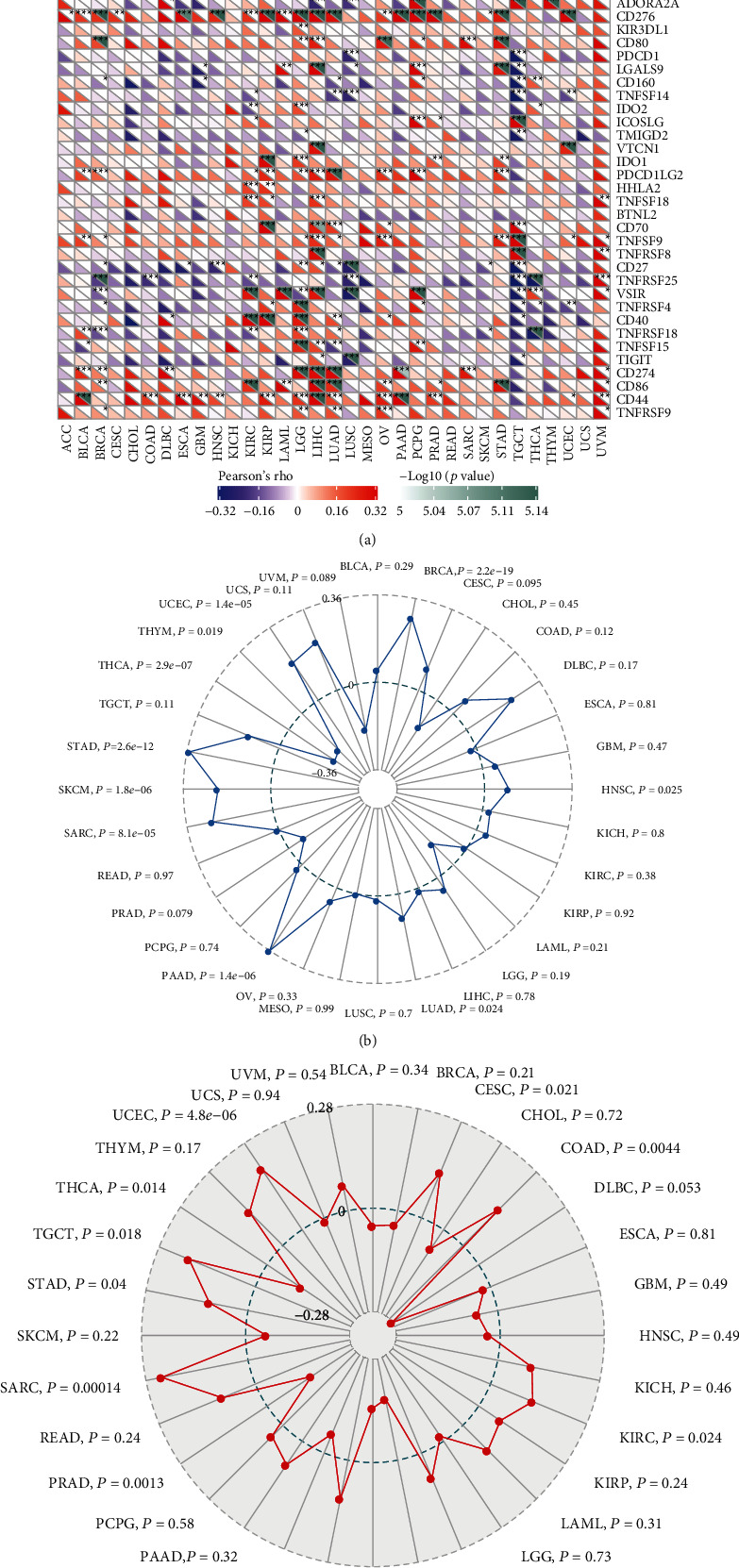
Analysis of association between PGK1 and immune checkpoints, TMB, and MSI across pan-cancer. (a) Heat map for the correlation between PGK1 and immune checkpoints across different cancer types with Pearson correlation analysis. Blue meant negative correlation while red meant positive correlation. ^∗^*p* < 0.05; ^∗∗^*p* < 0.01; ^∗∗∗^*p* < 0.001. (b) Association between PGK1 expression and tumor mutation burden (TMB) across pan-cancer utilizing Spearman correlation analysis. (c) Association between PGK1 expression and microsatellite instability (MSI) across pan-cancer with Spearman correlation analysis.

Association between PGK1 expression and immune checkpoints was analyzed at the mRNA levels across pan-cancer. In Figure 3(a), we observed that PGK1 was prominently associated with immune checkpoints across pan-cancer. Especially, PGK1 exhibited significant correlations to immune checkpoints including TNFRSF14, TNFSF4, CD40LG, HAVCR2, CD276, CD80, CD160, PDCD1LG2, TNFSF9, CD27, TNFRSF25, VSIR, TNFRSF4, TNFRSF18, CD274, CD86, and TNFRSF9. Furthermore, we found that PGK1 displayed significant correlations to TMB in BRCA (*p* = 2.2*e* − 19), HNSC (*p* = 0.025), LUAD (*p* = 0.024), PAAD (*p* = 1.4*e* − 06), SARC (*p* = 8.1*e* − 05), SKCM (*p* = 1.8*e* − 06), STAD (2.6*e* − 12), THCA (*p* = 2.9*e* − 07), THYM (*p* = 0.019), and UCEC (*p* = 1.4*e* − 05; Figure 3(b)). Association of PGK1 with MSI was then evaluated in different cancer types. In Figure 3(c), our results demonstrated the significant correlations between PGK1 expression and MSI in CESC (*p* = 0.021), COAD (*p* = 0.0044), KIRC (*p* = 0.024), LUAD (*p* = 0.00014), LUSC (*p* = 0.0011), PRAD (*p* = 0.0013), SARC (*p* = 0.00014), STAD (*p* = 0.04), TGCT (*p* = 0.018), THCA (*p* = 0.014), and UCEC (*p* = 4.8*e* − 06). Collectively, PGK1 could be in relation to immunotherapeutic responses.

### 3.3. PGK1 Acts as a Robust Risk Factor of Breast Cancer Outcomes

**Figure 4:**
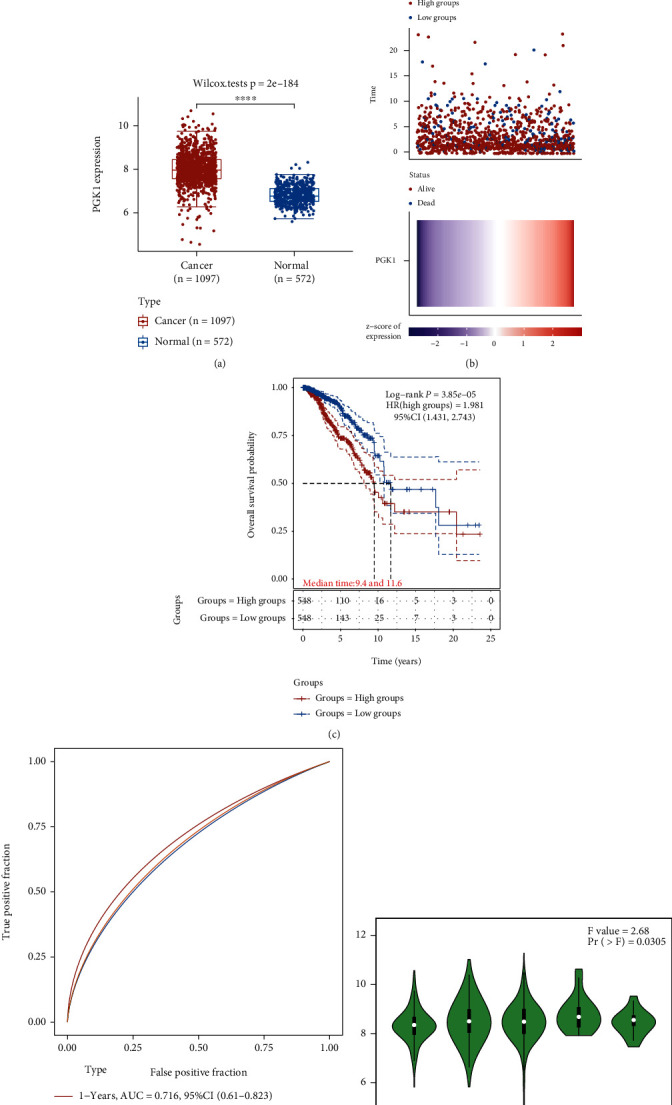
PGK1 acts as a robust risk factor of breast cancer prognosis. (a) Comparison of PGK1 expression in normal (*n* = 572) and breast cancer (*n* = 1097) tissues from TCGA cohort. The significant level was estimated with Wilcoxon rank-sum test. (b) Distribution of PGK1 expression (upper and bottom) and survival status (middle) in breast cancer patients from TCGA cohort. Breast cancer subjects were clustered into up- (red) and downregulated (blue) PGK1 subgroups. Red dots meant alive status while blue dots meant dead status. (c) Kaplan-Meier curves for estimating the survival difference in patients with high and low expression of PGK1. *p* value was calculated with log-rank test. (d) Time-independent ROC curve under one-, three-, and five-year survival for PGK1 expression across breast cancer patients. (e) Comparison of PGK1 expression across distinct pathological staging (stage I-X) using the GEPIA2 tool.

In the TCGA cohort, we compared the mRNA expression of PGK1 in normal (*n* = 572) and breast cancer (*n* = 1097) tissues. As a result, PGK1 expression exhibited marked upregulation in breast cancer in comparison to normal specimens (*p* = 2*e* − 184; Figure 4(a)). In line with the median value of PGK1 expression, we separated breast cancer subjects into two subgroups (Figure 4(b)). We observed that more patients had alive status in low expression subgroup. Survival difference between two groups was compared with log-rank test. As depicted in Figure 4(c), patients with high PGK1 expression (median time = 9.4) were indicative of unfavorable clinical outcomes than those with low PGK1 expression (median time = 11.6; *p* = 3.85*e* − 05). ROC curves were conducted for assessing the performance of PGK1 in prediction of breast cancer prognosis (Figure 4(d)). AUC values under 1-, 3-, and 5-year survival were 0.716, 0.682, and 0.678, indicative of PGK1 as a robust predictor. Using the GEPIA2 tool, we evaluated the expression of PGK1 mRNA across distinct pathological stages (stage I-X) across breast cancer patients. Our results showed that PGK1 displayed the highest mRNA expression in stage IV (*p* = 0.0305; Figure 4(e)). Taken together, PGK1 may act as a reliable risk factor of breast cancer prognosis.

### 3.4. Association between PGK1 and Clinical Phenotype of Breast Cancer

**Figure 5:**
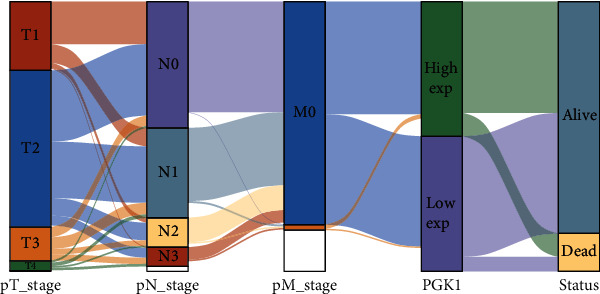
Analysis of association between PGK1 and clinical phenotype of breast cancer in TCGA dataset. Sankey diagram depicted the distribution alterations of pathological T stage (T1-4), pathological N stage (N0-3), pathological M stage (M0 and M1), PGK1 expression (high and low expression), and survival status (alive and dead). Each column represented a clinical phenotype. Different colors indicated different clinical phenotypes. The lines reflected the distribution of the same sample in different characteristic variables.

Herein, we evaluated the significant associations between PGK1 and clinical phenotypes (pathological T stage (T1-4), pathological N stage (N0-3), pathological M stage (M0 and M1), and survival status (alive and dead)) of breast cancer patients in TCGA dataset, as shown in Sankey diagram (Figure 5).

### 3.5. Association between PGK1 and Immune Infiltrates

**Figure 6:**
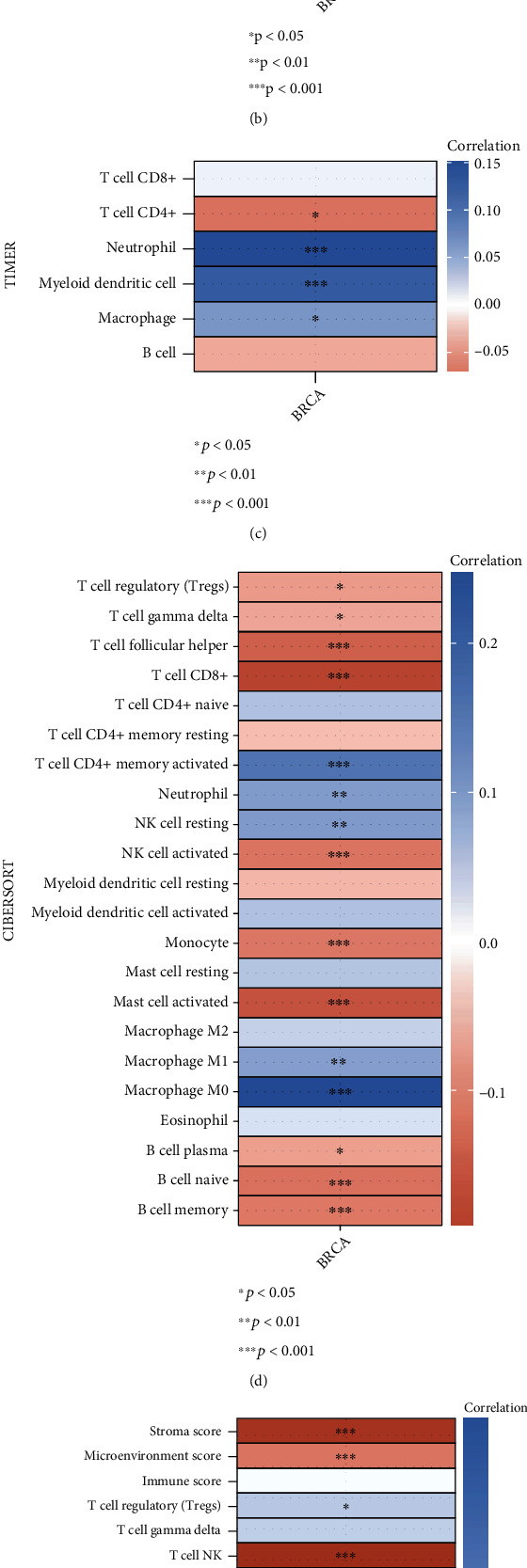
Integrated analysis of associations between PGK1 and immune cell subpopulations in breast cancer specimens from TCGA dataset. (a) Heat map depicting the correlation of PGK1 with the abundance levels of lymphocyte subpopulations through MCP-counter algorithm. (b) Heat map for the correlation of PGK1 with the abundance levels of lymphocyte subpopulations with quanTIseq algorithm. (c) Heat map for the associations between PGK1 expression and the abundance levels of lymphocyte subpopulations using TIMER algorithm. (d) Heat map showing the associations between PGK1 expression and the abundance levels of immune cell subpopulations utilizing CIBERSORT algorithm. (e) Heat map demonstrating the associations between PGK1 expression and the abundance levels of lymphocyte subpopulations with xCell algorithm. Red meant positive correlation and blue meant negative correlation. The darker the color, the stronger the correlation. ^∗^*p* < 0.05; ^∗∗^*p* < 0.01; ^∗∗∗^*p* < 0.001.

Immunedeconv method was used for analyzing the abundance levels of immune cells subpopulations across breast cancer samples in the TCGA cohort, including MCP-counter, quanTIseq, CIBERSORT, xCell, and TIMER algorithms. The associations between PGK1 and immune cell subpopulations were estimated via Spearman correlation analysis. For the MCP-counter algorithm, we observed that PGK1 expression was positively associated with T cell, myeloid dendritic cell, endothelial cell, and B cell but negatively associated with monocyte and macrophage (Figure 6(a)). For the quanTIseq algorithm, PGK1 expression displayed positive correlation to T cell CD4+, NK cell, and macrophage M2 but exhibited negative correlation to T cell regulatory (Treg), neutrophil, and macrophage M1 (Figure 6(b)). For the TIMER algorithm, PGK1 expression displayed a positive association with T cell CD4+ but negative associations with neutrophil, myeloid dendritic cell, and macrophage (Figure 6(c)). For the CIBERSORT algorithm, PGK1 was positively correlated to Treg, T cell gamma delta, T cell follicular helper, T cell CD8+, NK cell activated, monocyte, mast cell activated, B cell plasma, B cell native, and B cell memory (Figure 6(d)). In contrast, there were negative correlations between PGK1 expression and T cell CD4+ memory activated, neutrophil, NK cell resting, macrophage M1, and macrophage M0. For the xCell algorithm, PGK1 expression had positive correlations to stromal score, microenvironment score, T cell NK, T cell CD4+ naïve, T cell CD4+ effector memory, T cell CD4+ central memory, macrophage M2, hematopoietic stem cell, endothelial cell, common myeloid progenitor, and B cell memory but had negative correlations to T cell CD4+ memory, T cell CD4+ Th2, plasmacytoid dendritic cell, NK cell, monocyte, macrophage M1, macrophage, and common lymphoid progenitor (Figure 6(e)). Collectively, PGK1 was in relation to an inflamed microenvironment in breast cancer.

### 3.6. Biological Pathways and Hallmarks of Cancer Involved in PGK1

**Figure 7:**
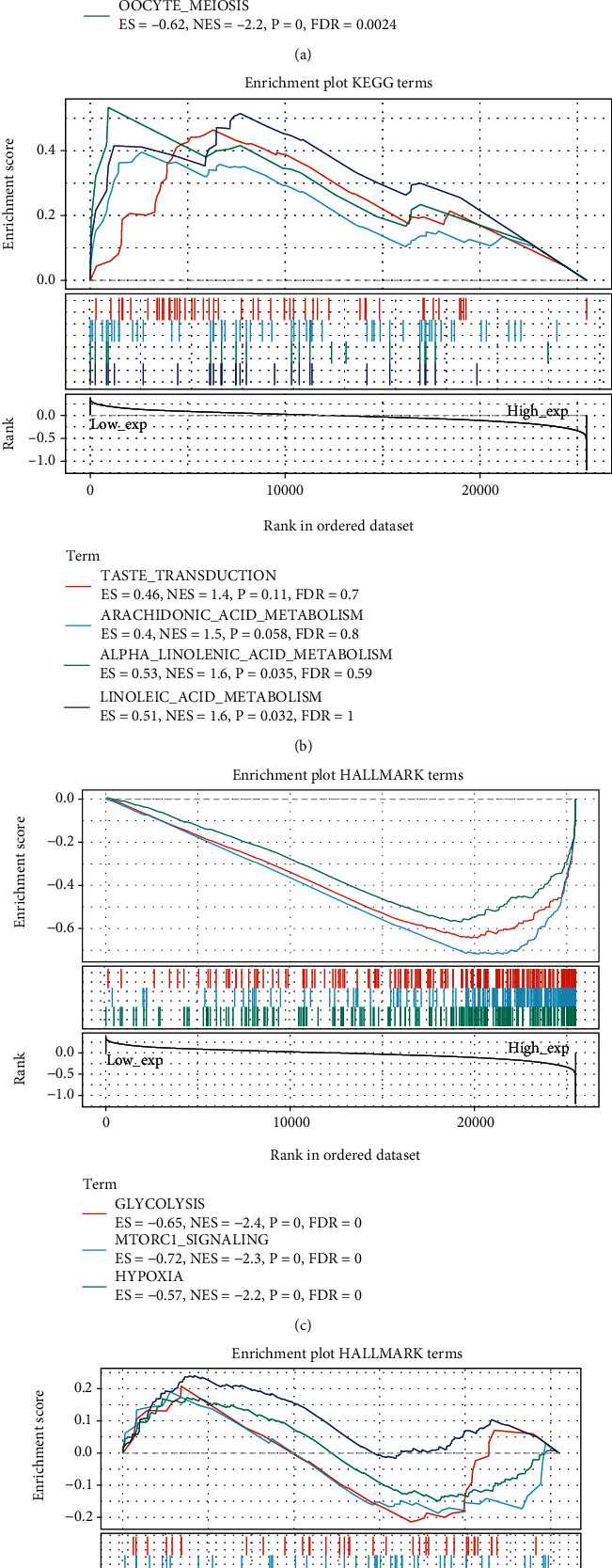
GSEA identifies biological pathways and hallmarks of cancer involved in PGK1. (a) and (b) KEGG pathways involved in PGK1 through comparison of up- and downregulated PGK1 groups across breast cancer samples from TCGA dataset. (c) and (d) Hallmarks of cancer involved in PGK1 via comparison of up- and downregulated PGK1 groups across breast cancer samples from TCGA dataset. The enrichment score (ES) reflected the degree to which the indicated gene set was overrepresented at the extreme (top or bottom) of the whole ordered gene list.

Through GSEA method, we investigated biological pathways and hallmarks of cancer involved in PGK1 through comparison of up- and downregulated PGK1 groups across breast cancer samples from the TCGA dataset. Our results showed that pyrimidine metabolism (ES = −0.67, NES = −2.4, *p* < 0.0001, FDR = 0.001), cell cycle (ES = −0.72, NES = −2.3, *p* < 0.0001, FDR = 0.0023), and oocyte meiosis (ES = −0.62, NES = −2.2, *p* < 0.0001, FDR = 0.0024) pathways were negatively correlated to PGK1 expression (Figure 7(a)). Meanwhile, taste transduction (ES = 0.46, NES = 1.4, *p* = 0.11, FDR = 0.7), arachidonic acid metabolism (ES = 0.4, NES = 1.5, *p* = 0.058, FDR = 0.8), alpha linolenic acid metabolism (ES = 0.53, NES = 1.6, *p* = 0.035, FDR = 0.59), and linoleic acid metabolism (ES = 0.51, NES = 1.6, *p* = 0.032, FDR = 1) displayed positive correlations to PGK1 expression (Figure 7(b)). In Figures 7(c) and 7(d), PGK1 expression exhibited associations with hallmarks of cancer including glycolysis (ES = −0.65, NES = −2.4, *p* < 0.0001, FDR < 0.0001), mTORC1 signaling (ES = −0.72, NES = −2.3, *p* < 0.0001, FDR < 0.0001), hypoxia (ES = −0.57, NES = −2.2, *p* < 0.0001, FDR < 0.0001), Hedgehog signaling (ES = −0.23, NES = −0.77, *p* = 0.75, FDR = 0.7), Wnt *β*-catenin signaling (ES = −0.22, NES = −0.72, *p* = 0.8, FDR = 0.75), myogenesis (ES = 0.17, NES = 0.69, *p* = 0.9, FDR = 0.8), and KRAS signaling (ES = 0.24, NES = 1.1, *p* = 0.35, FDR = 0.65).

## 4. Discussion

In this study, we elucidated that PGK1 shaped an inflamed tumor microenvironment in line with the evidence that PGK1 displayed positive correlations to the immunological status of tumor microenvironment in breast cancer. Furthermore, this study proposed that PGK1 deregulation was capable of robustly predicting survival outcomes as well as immunotherapeutic responses.

Our pan-cancer analysis uncovered that PGK1 upregulation contributed to undesirable clinical outcomes for most of cancer types. ROC curves confirmed the favorable performance in prediction of breast cancer prognosis. This demonstrated that PGK1 possessed the potential as a reliable prognostic predictor of breast cancer. Patients with high TMB can benefit from immunotherapeutic agents. Preliminary evidence suggests that hypermutation of breast cancer is more likely to benefit from anti-PD-1 therapy [34]. Furthermore, evidence demonstrates prominent response of cancers with MSI to anti-PD-1 therapy for patients who failed conventional treatment [35]. Herein, we observed the positive associations of PGK1 with TMB and MSI across pan-cancer, especially breast cancer, indicative of the potential of PGK1 as an immune response predictor. Through six algorithms including MCP-counter, quanTIseq, CIBERSORT, xCell, and TIMER, we estimated the associations of PGK1 with the infiltration levels of lymphocytes across breast cancer. We observed that PGK1 displayed positive correlations to CD8+ T cells and activated NK cells as well as was prominently associated with immune checkpoints. This indicated that PGK1 might shape an inflamed phenotype of tumor microenvironment in breast cancer.

Our GSEA identified that PGK1 was in relation to KEGG pathways including pyrimidine metabolism, cell cycle, oocyte meiosis pathways, taste transduction, arachidonic acid metabolism, alpha-linolenic acid metabolism, and linoleic acid metabolism. Furthermore, we found that PGK1 displayed correlations to hallmarks of cancer including glycolysis, mTORC1 signaling, hypoxia, Hedgehog signaling, Wnt *β*-catenin signaling, myogenesis, and KRAS signaling. Previous experiments have confirmed the regulatory roles of PGK1 on above pathways. For instance, PGK1 inhibition could counteract chemoresistance to intraperitoneal 5-fluorouracil in gastric cancer [36]. Nuclear PGK1 reduces ADP-dependent suppression of CDC7 to enhance DNA replication [37]. PGK1-coupled HSP90 may stabilize GSK3*β* expression to modulate the stemness features in breast cancer [38].

Nevertheless, there are limitations in our study. First, we cannot identify the optimal cut-off of PGK1 among breast cancer patients. Hence, the median mRNA expression of PGK1 was chosen as the cut-off. Second, in-depth experiments will be presented for determining the expression profiling of PGK1 in breast cancer cells as well as tumor-infiltrating immune cells. Third, the predictive performance of PGK1 should be externally validated in a larger cohort. Fourth, in vitro and in vivo experiments will be performed to explore the potential function of PGK1 dysregulation in the proliferation, migration, and invasion of breast cancer.

## 5. Conclusion

This study suggested that PGK1 may act as a suitable candidate for the prediction of breast cancer prognosis. Moreover, our findings indicated that PGK1 shaped an inflamed tumor microenvironment in breast cancer as well as could predict the clinical response to immunotherapy.

## Data Availability

The datasets analyzed during the current study are available from the corresponding author on reasonable request.

## Abbreviations

PGK1: Phosphoglycerate kinase 1
ICIs: Immune checkpoint inhibitors
TIMER: Tumor Immune Estimation Resource
TCGA: The Cancer Genome Atlas
TPM: Transcripts Per Million
RNA-seq: RNA sequencing
GDC: Genomic Data Commons
HR: Hazard ratio
CI: Confidence interval
OS: Overall survival
DFI: Disease-free interval
DSS: Disease-specific survival
PFI: Progression-free interval
GEPIA2: Gene Expression Profiling Interactive Analysis 2
GTEx: Genotype-Tissue Expression
TMB: Tumor mutation burden
MSI: Microsatellite instability
MCP-counter: Microenvironment Cell Populations-counter
CIBERSORT: Cell type Identification By Estimating Relative Subsets Of RNA Transcripts
GSEA: Gene set enrichment analyses
ES: Enrichment score
KEGG: Kyoto Encyclopedia of Genes and Genomes
MSigDB: Molecular Signature Database
NES: Normalized enrichment score
FDR: False discovery rate.

## References

[1] Bray F., Ferlay J., Soerjomataram I., Siegel R. L., Torre L. A., Jemal A. (2018). Global cancer statistics 2018: GLOBOCAN estimates of incidence and mortality worldwide for 36 cancers in 185 countries. *CA: a Cancer Journal for Clinicians*.

[2] Morgan A. J., Giannoudis A., Palmieri C. (2021). The genomic landscape of breast cancer brain metastases: a systematic review. *The Lancet Oncology*.

[3] Parsons H. A., Burstein H. J. (2021). Adjuvant capecitabine in triple-negative breast Cancer. *JAMA*.

[4] Pusztai L. (2021). PARP inhibition in homologous-recombination-deficient early-stage breast cancer. *Annals of Oncology*.

[5] Krug K., Jaehnig E. J., Satpathy S. (2020). Proteogenomic landscape of breast cancer tumorigenesis and targeted therapy. *Cell*.

[6] Vonderheide R. H., LoRusso P. M., Khalil M. (2010). Tremelimumab in combination with exemestane in patients with advanced breast cancer and treatment-associated modulation of inducible costimulator expression on patient T cells. *Clinical Cancer Research*.

[7] Fumet J. D., Limagne E., Thibaudin M. (2020). Precision medicine phase II study evaluating the efficacy of a double immunotherapy by durvalumab and tremelimumab combined with olaparib in patients with solid cancers and carriers of homologous recombination repair genes mutation in response or stable after olaparib treatment. *BMC Cancer*.

[8] Voorwerk L., Slagter M., Horlings H. M. (2019). Immune induction strategies in metastatic triple-negative breast cancer to enhance the sensitivity to PD-1 blockade: the TONIC trial. *Nature Medicine*.

[9] Esteva F. J., Hubbard-Lucey V. M., Tang J., Pusztai L. (2019). Immunotherapy and targeted therapy combinations in metastatic breast cancer. *The Lancet Oncology*.

[10] Gil Del Alcazar C. R., Alečković M., Polyak K. (2020). Immune escape during breast tumor progression. *Cancer Immunology Research*.

[11] Keenan T. E., Tolaney S. M. (2020). Role of immunotherapy in triple-negative breast cancer. *Journal of the National Comprehensive Cancer Network*.

[12] Franzoi M. A., Romano E., Piccart M. (2021). Immunotherapy for early breast cancer: too soon, too superficial, or just right?. *Annals of Oncology*.

[13] Stanton S. E., Disis M. L. (2016). Clinical significance of tumor-infiltrating lymphocytes in breast cancer. *Journal for Immunotherapy of Cancer*.

[14] van Weverwijk A., Koundouros N., Iravani M. (2019). Metabolic adaptability in metastatic breast cancer by AKR1B10-dependent balancing of glycolysis and fatty acid oxidation. *Nature Communications*.

[15] Chu Z., Huo N., Zhu X. (2021). FOXO3A-induced LINC00926 suppresses breast tumor growth and metastasis through inhibition of PGK1-mediated Warburg effect. *Molecular Therapy*.

[16] Li X., Jiang Y., Meisenhelder J. (2016). Mitochondria-translocated PGK1 functions as a protein kinase to coordinate glycolysis and the TCA cycle in tumorigenesis. *Molecular Cell*.

[17] Fu Q., Yu Z. (2020). Phosphoglycerate kinase 1 (PGK1) in cancer: a promising target for diagnosis and therapy. *Life Sciences*.

[18] He Y., Luo Y., Zhang D. (2019). PGK1-mediated cancer progression and drug resistance. *American Journal of Cancer Research*.

[19] Liang C., Shi S., Qin Y. (2020). Localisation of PGK1 determines metabolic phenotype to balance metastasis and proliferation in patients with SMAD4-negative pancreatic cancer. *Gut*.

[20] Nie H., Ju H., Fan J. (2020). O-GlcNAcylation of PGK1 coordinates glycolysis and TCA cycle to promote tumor growth. *Nature Communications*.

[21] Feng X., Zhang H., Meng L. (2021). Hypoxia-induced acetylation of PAK1 enhances autophagy and promotes brain tumorigenesis via phosphorylating ATG5. *Autophagy*.

[22] Li T., Fu J., Zeng Z. (2020). TIMER2.0 for analysis of tumor-infiltrating immune cells. *Nucleic Acids Research*.

[23] Weinstein J. N., Collisson E. A., Mills G. B. (2013). The cancer genome atlas pan-cancer analysis project. *Nature Genetics*.

[24] Fancello L., Gandini S., Pelicci P. G., Mazzarella L. (2019). Tumor mutational burden quantification from targeted gene panels: major advancements and challenges. *Journal for Immunotherapy of Cancer*.

[25] Hause R. J., Pritchard C. C., Shendure J., Salipante S. J. (2016). Classification and characterization of microsatellite instability across 18 cancer types. *Nature Medicine*.

[26] Tang Z., Kang B., Li C., Chen T., Zhang Z. (2019). GEPIA2: an enhanced web server for large-scale expression profiling and interactive analysis. *Nucleic Acids Research*.

[27] Sturm G., Finotello F., List M. (2020). Immunedeconv: an R package for unified access to computational methods for estimating immune cell fractions from bulk RNA-sequencing data. *Methods in Molecular Biology*.

[28] Becht E., Giraldo N. A., Lacroix L. (2016). Estimating the population abundance of tissue-infiltrating immune and stromal cell populations using gene expression. *Genome Biology*.

[29] Finotello F., Mayer C., Plattner C. (2019). Molecular and pharmacological modulators of the tumor immune contexture revealed by deconvolution of RNA-seq data. *Genome Medicine*.

[30] Newman A. M., Liu C. L., Green M. R. (2015). Robust enumeration of cell subsets from tissue expression profiles. *Nature Methods*.

[31] Aran D., Hu Z., Butte A. J. (2017). xCell: digitally portraying the tissue cellular heterogeneity landscape. *Genome Biology*.

[32] Subramanian A., Tamayo P., Mootha V. K. (2005). Gene set enrichment analysis: a knowledge-based approach for interpreting genome-wide expression profiles. *Proceedings of the National Academy of Sciences of the United States of America*.

[33] Liberzon A., Birger C., Thorvaldsdóttir H., Ghandi M., Mesirov J. . P., Tamayo P. (2015). The Molecular Signatures Database Hallmark Gene Set Collection. *Cell Systems*.

[34] Barroso-Sousa R., Jain E., Cohen O. (2020). Prevalence and mutational determinants of high tumor mutation burden in breast cancer. *Annals of Oncology*.

[35] Dudley J. C., Lin M. T., Le D. T., Eshleman J. R. (2016). Microsatellite instability as a biomarker for PD-1 blockade. *Clinical Cancer Research*.

[36] Archid R., Zieker D., Weinreich F. J. (2020). shRNA-mediated inhibition of phosphoGlycerate kinase 1 (PGK1) enhances cytotoxicity of intraperitoneal chemotherapy in peritoneal metastasis of gastric origin. *European Journal of Surgical Oncology*.

[37] Li X., Qian X., Jiang H. (2018). Nuclear PGK1 alleviates ADP-dependent inhibition of CDC7 to promote DNA replication. *Molecular cell*.

[38] Tang W., Wu Y., Qi X. (2021). PGK1-coupled HSP90 stabilizes GSK3*β* expression to regulate the stemness of breast cancer stem cells. *Cancer Biology & Medicine*.

